# Prognostic value and underlying mechanism of KIAA0101 in hepatocellular carcinoma: database mining and co-expression analysis

**DOI:** 10.18632/aging.103704

**Published:** 2020-08-27

**Authors:** Weiyu Xu, Xuezhu Wang, Xiaoqian Wu, Si Yu, Jianping Xiong, Xinting Sang, Yongchang Zheng, Zhongtao Zhang

**Affiliations:** 1 Department of General Surgery, Beijing Friendship Hospital, Capital Medical University, Beijing 100050, China; 2 Department of Liver Surgery, Peking Union Medical College Hospital, Chinese Academy of Medical Sciences and Peking Union Medical College (CAMS and PUMC), Beijing 100730, China; 3 Department of Interventional Radiology, Beijing Friendship Hospital, Capital Medical University, Beijing 100050, China

**Keywords:** KIAA0101, hepatocellular carcinoma, prognosis, co-expression network, database mining

## Abstract

Although KIAA0101 is involved in many diseases, its expression and prognostic value in HCC remain undefined. According to CCLE, KIAA0101 is highly expressed in HCC, with a weak positive correlation between copy number and gene expression. Four studies involving 760 samples in ONCOMINE report elevated KIAA0101 expression in HCC (p=3.11E-22). The KM plotter revealed high KIAA0101 expression to be associated with worse overall survival in HCC (HR=2.09, p=4.1e-05); this prognostic power was stronger for male than female, early-stage than advanced-stage, and Asian than Caucasian patients. RNA sequencing data for 8 pairs of HCC and adjacent tissue samples validated the significantly high KIAA0101 level (p=0.00497). Moreover, functional annotations of 31 KIAA0101-coexpressed genes show enrichment of terms associated with mitosis, cytoskeleton construction, and chromosome segregation. Among 9 genes having STRING-validated protein-protein interactions with KIAA0101, two are involved in virus-related pathways. Alternative splicing analysis indicated higher expression of variant 1 and variant 2 in HCC and no significant differences in exon usage of KIAA0101 between cancer and normal tissues. These findings support that KIAA0101 is a potential prognostic biomarker for HCC and highlight the association between virus infection and the mechanism underlying the process by which KIAA0101 contributes to poor prognosis of patients.

## INTRODUCTION

Hepatocellular carcinoma (HCC), a well-defined phenotypically and molecularly heterogeneous disease, is the second most lethal malignancy globally [1]. The carcinogenesis of HCC is a complicated, multistep process involving multiple changes to signaling cascades, resulting in heterogeneous molecular profiles and initiation-promotion-progression [2]. Despite great efforts to enhance diverse therapeutic techniques (including surgical dissection, liver transplantation and ablation), which can temporarily improve HCC patients’ quality of life regardless of their stage, the prognosis of HCC is still unsatisfactory [3, 4]. The absence of sensitive screening examinations has contributed to the difficulty of early diagnosis of the disease. Moreover, only 30–40% of HCC patients can be cured after diagnosis [5]. Tumor node metastasis (TNM) stage and pathological grade are widely used for prognosis, yet challenges remain in accurately staging and grading tumors, and substaging remains controversial [6]. In clinical settings, several HCC biomarkers have been determined to predict prognosis, including AFP [7], CEA [8], and CA 19-9 [9], all of which are traditional tumor markers. Regardless, none of these biomarkers is ideal or applicable because of their low sensitivity and/or specificity in clinical diagnosis. Therefore, it is necessary to identify specific prognostic markers, regardless of hepatic function and diseases with different etiologies, to guide HCC therapy and improve the overall survival (OS) of these patients.

KIAA0101, also known as P15^PAF^ (PCNA-associated factor) [10], OEATC-1 (overexpressed in anaplastic thyroid carcinoma-1)[11], and L5 [12], is a 15-kDa protein that contains a conserved proliferating cell nuclear antigen (PCNA)-binding motif [13, 14]. PCNA is not only a vital factor for DNA polymerase, which is necessary for DNA replication and repair [15, 16], but it can also bind to other PCNA-binding proteins, including p33ING1b, p57^Kip2^ and p21^WAF^, to promote DNA synthesis [17], cell cycle progression [18, 19], and G_1_ cell cycle arrest [20]. Thus, KIAA0101 is closely associated with DNA repair [21], cell cycle progression [21] and cell proliferation [10].

Based on the above studies, KIAA0101 might be applied as an important and ideal predictive and prognostic marker for human cancer. Actually, in recent years, several studies have found that KIAA0101 is overexpressed in various cancers, such as breast cancer [22], glioma [23], renal cancer [24], pancreatic cancer [25], esophageal cancer [26], lung small-cell cancer [27], colorectal cancer [28], and HCC [29, 30], and is related to a poor outcome. However, previous studies involving the prognostic value of KIAA0101 in HCC utilized different methods, sample sizes, and populations, which undermines the statistical power of the results and makes the conclusions less reliable. Moreover, the mechanism underlying the prognostic value of KIAA0101 in cancer remains ambiguous, and this aspect is critical because it can influence the prognostic strength of KIAA0101 for different medical conditions.

Therefore, the current study involves an overall investigation of the differential expression and prognostic significance of KIAA0101 in HCC, with the aim of identifying factors that influence its prognostic value and studying the potential and causative mechanism by coexpression analysis.

## RESULTS

### CCLE shows a correlation between KIAA0101 expression and copy number variation in cancer cell lines, including liver cancer

**Figure 1:**
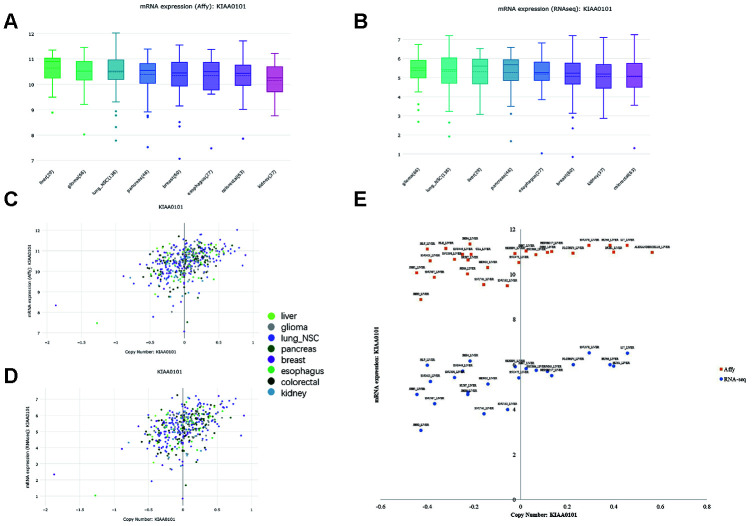
**KIAA0101 mRNA expression and copy number in the CCLE database. In the box plots, the solid line indicates the median value, and the dashed line indicates the mean value.** (**A**) KIAA0101 mRNA expression (Affy) levels in liver cancer, glioma, lung nonsmall-cell cancer, pancreatic cancer, breast cancer, esophageal cancer, colorectal cancer, and kidney cancer. The highest KIAA0101 expression was observed in liver cancer. (**B**) KIAA0101 mRNA expression (RNA-seq) level in these cancer cell lines. (**C**) The scatter plot demonstrating KIAA0101 mRNA expression (Affy) level and KIAA0101 copy number of cancer cell lines, including liver cancer, glioma, lung nonsmall-cell cancer, pancreatic cancer, breast cancer, esophageal cancer, colorectal cancer, and kidney cancer. (**D**) The scatter plot demonstrating KIAA0101 mRNA expression (RNA-seq) level and KIAA0101 copy number of these cancer cell lines. (**E**) The scatter plot demonstrating the KIAA0101 mRNA expression level and KIAA0101 copy number of different cancer cell lines. Orange squares indicate mRNA levels quantified by Affymetrix; blue circles indicate mRNA expression profiled by RNA-seq. The names of these liver cancer cell lines are annotated, including ALEXANDERCELLS_LIVER, C3A_LIVER, HEP3B217_LIVER, HEPG2_LIVER, HLE_LIVER, HLF_LIVER, HUH1_LIVER, HUH6_LIVER, HUH7_LIVER, JHH1_LIVER, JHH2_LIVER, JHH4_LIVER, JHH5_LIVER, JHH6_LIVER, JHH7_LIVER, LI7_LIVER, NCIH684_LIVER, PLCPRF5_LIVER, SKHEP1_LIVER, SNU182_LIVER, SNU387_LIVER, SNU398_LIVER, SNU423_LIVER, SNU449_LIVER, SNU475_LIVER, SNU761_LIVER, SNU878_LIVER and SNU886_LIVER.

The CCLE database offers a large dataset of gene expression and copy number variation (CNV) in cancer cell lines [34]. However, due to a lack of normal counterparts for comparison, cancers with reported KIAA0101 overexpression and prognostic value are included together with liver cancer. KIAA0101 expression levels in liver cancer, glioma, lung nonsmall-cell cancer, pancreatic cancer, breast cancer, esophageal cancer, colorectal cancer, and kidney cancer exhibited an insignificant difference based on both Affymetrix and RNA-seq profiling (Figure 1A and 1B). The copy number of KIAA0101 approximately correlated positively with KIAA0101 expression, but the strength of the correlation varied in different cancer cell lines (Figure 1C and 1D). Specifically, the correlation was relatively weak in the liver cancer cell line. In cell lines with a KIAA0101 copy number higher than 0, expression of the gene tended to be high as well; however, when the KIAA0101 copy number was below 0, its expression was equally high or significantly low (Figure 1E). These results indicate that factors in addition to CNV are likely to contribute to KIAA0101 overexpression in HCC. In addition, Affymetrix and RNA-seq profiling demonstrated a similar pattern of correlation between gene expression and copy number for KIAA0101.

### ONCOMINE reveals the mRNA KIAA0101 level in human cancers, including HCC

**Figure 2:**
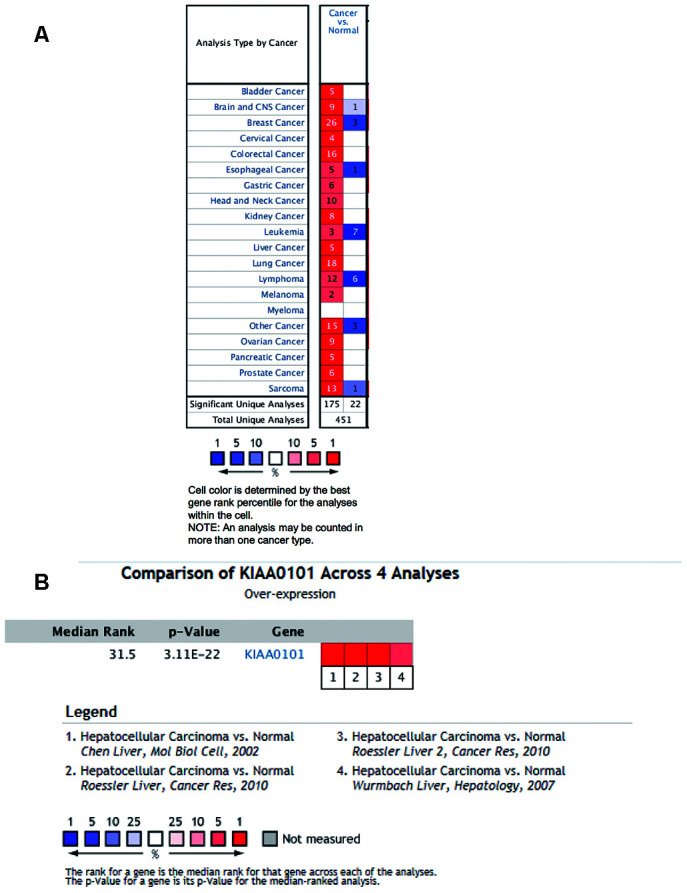
**Ranking mRNA expression levels of KIAA0101 in a variety of cancers in the database ONCOMINE.** (**A**) The number in the colored cell represents the number of analyses meeting thresholds. The cell color is determined by the best gene rank percentile for the analyses within the cell. The more intense red (overexpression) or blue (underexpression) indicates a more highly significant overexpressed or underexpressed gene. (**B**) mRNA expression of KIAA0101 in HCC demonstrated by 4 unique analyses in the ONCOMINE database: the Chen liver, the Roessler liver, the Roessler liver 2, and the Wurmbach liver datasets. The median rank for KIAA0110 is across each of the analyses. The p-value is for the media-ranked analysis. Darker red indicates a higher best gene rank percentile for the analyses within the cell. Abbreviation: HCC: hepatocellular carcinoma.

**Figure 3:**
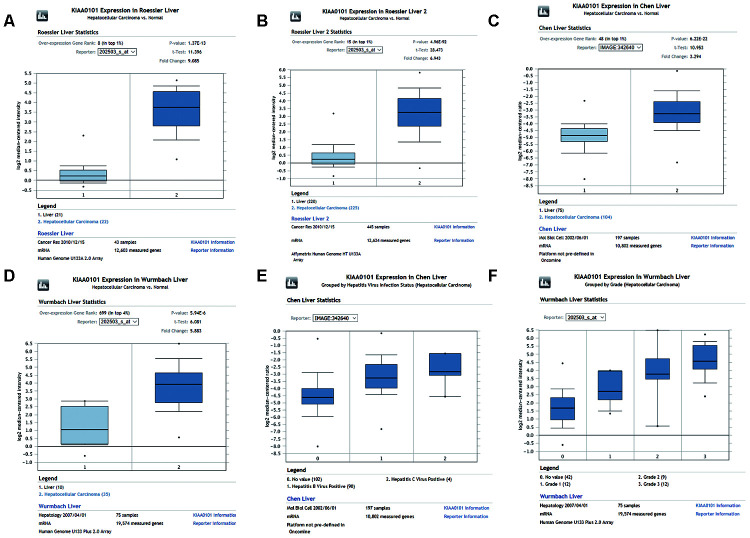
**Comparison of KIAA0101 mRNA levels between HCC and normal counterparts by the four unique analyses of ONCOMINE.** (**A**) Comparison of KIAA0101 mRNA levels between noncancer tissues and HCC tumors in Roessler’s study based on Human Genome U133A 2.0 Array in the ONCOMINE database. KIAA0101 presented significantly higher expression in HCC tissues than in noncancer tissues (p=1.37E-13). (**B**) Comparison of KIAA0101 mRNA levels between noncancer tissues and HCC tumors in Roessler’s study based on the platform of Affymetrix Human Genome HT U133A Array in the ONCOMINE database. KIAA0101 expression was significantly higher in HCC tissues than in noncancer tissues (p=4.96E-92). (**C**) Comparison of KIAA0101 mRNA levels between noncancer tissues and HCC tumors in Chen’s study in the ONCOMINE database. KIAA0101 presented significantly higher expression in HCC tissues than in noncancer tissues (p= 6.22E-22). (**D**) Comparison of KIAA0101 mRNA levels between noncancer tissues and HCC tumors in Wurmbach’s study, which was identified in the ONCOMINE database. KIAA0101 presented significantly higher expression in HCC tissues than in noncancer tissues (p= 5.94E-6). (**E**) KIAA0101 expression in HCC grouped by virus infection status. The HCC samples were no value (102 samples), HBV positive (90 samples), and HCV positive (4 samples) in Chen’s liver dataset. (**F**) KIAA0101 expression in HCC grouped by tumor grade. The HCC samples are no value (42 samples), grade 1 (12 samples), grade 2 (9 samples), and grade 3 (12 samples) in Wurmbach’s liver dataset. Abbreviations: HCC: hepatocellular carcinoma; HBV: hepatitis B virus; HCV: hepatitis C virus.

We investigated differences in mRNA expression of KIAA0101 between cancer tissues and normal tissues in human solid tumors and hematological malignancies. Of 451 unique analyses in the ONCOMINE database, there were 197 datasets showing statistically significant differences in KIAA0101 expression ranking among the top 10% of all genes; 175 studies demonstrated higher expression of KIAA0101 in cancer than in normal tissues, whereas 22 studies found the opposite results. All five unique analyses of liver cancer reported KIAA0101 overexpression ranking in the top 10% (Figure 2A). A meta-analysis of KIAA0101 overexpression in the four unique analyses of HCC was performed. Compared with the normal tissue group, the median rank for KIAA0101 was 31.5 (p= 3.11E-22), which strongly suggested KIAA0101 overexpression in HCC (Figure 2B). In addition, the dataset originating from Roessler’s study, which used the platform of Human Genome U133A 2.0 Array, reported a 9.085-fold elevation of KIAA0101 transcripts in HCC specimens in comparison to normal tissues (p=1.37E-13) (N = 43) (Figure 3A). Another study by Roessler [33] employed the Affymetrix Human Genome HT U133A Array platform, finding a 6.943-fold increase in KIAA0101 transcripts in HCC specimens compared with normal tissues (p=4.96E-92) in a dataset comprising 445 samples (Figure 3B). The dataset for Chen’s study [32] with 197 samples indicated a 3.294-fold elevation of KIAA0101 in HCC specimens compared with normal tissues (p= 6.22E-22) (Figure 3C). KIAA0101 also showed a 5.883-fold increase in HCC specimens in comparison to normal tissues in the study conducted by Wurmbach [31] (p= 5.94E-6), a dataset of 75 samples (Figure 3D). In addition to overexpression of KIAA0101 in HCC, ONCOMINE revealed an association between the KIAA0101 level, virus infection status, and tumor grade. According to the Chen liver dataset [32], HCV-positive HCC had the highest level of KIAA0101, followed by HBV-positive HCC and no-value samples (Figure 3E). For the Wurmbach liver dataset [31], the grades 1, 2, and 3 HCC datasets were of similar size, and KIAA0101 expression prominently increased with tumor grade (Figure 3F). These associations indicate that the virus infection status and tumor grade may correlate with the prognostic value of KIAA0101.

### KIAA0101 shows different prognostic value in different HCC subgroups

**Figure 4:**
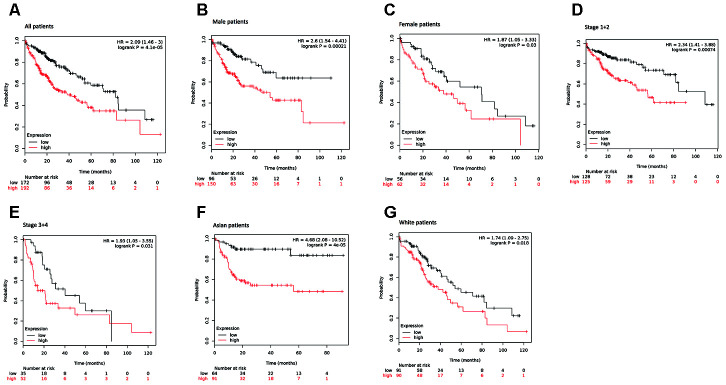
**Survival analyses of KIAA0101 mRNA expression levels in HCC patients.** (**A**) Survival analyses of KIAA0101 mRNA expression levels in HCC based on the Kaplan–Meier plotter database. Kaplan–Meier survival curves showed significant differences in OS between HCC patients with high and low KIAA0101 mRNA expression levels (HR=2.09, p=4.1e-05, log-rank test). (**B**) Survival analyses of KIAA0101 mRNA expression levels in HCC based on the Kaplan–Meier plotter database. Kaplan–Meier survival curves showed significant differences in OS between male HCC patients with high and low KIAA0101 mRNA expression levels (HR=2.6, p=0.00021, log-rank test). (**C**) Survival analyses of KIAA0101 mRNA expression levels in HCC based on the Kaplan–Meier plotter database. Kaplan–Meier survival curves showed significant differences in OS between female HCC patients with high and low KIAA0101 mRNA expression levels (HR=1.87, p=0.03, log-rank test). (**D**) Survival analyses of KIAA0101 mRNA expression levels in HCC based on the Kaplan–Meier plotter database. Kaplan–Meier survival curves showed significant differences in OS between early-stage (stage 1+2) HCC patients with high and low KIAA0101 mRNA expression levels (HR=2.34 p=0.00074, log-rank test). (**E**) Survival analyses of KIAA0101 mRNA expression levels in HCC based on the Kaplan–Meier plotter database. Kaplan–Meier survival curves showed significant differences in OS between advanced-stage (stage 3+4) HCC patients with high and low KIAA0101 mRNA expression levels (HR=1.93, p=0.031, log-rank test). (**F**) Survival analyses of KIAA0101 mRNA expression levels in HCC based on the Kaplan–Meier plotter database. Kaplan–Meier survival curves showed significant differences in OS between Asian HCC patients with high and low KIAA0101 mRNA expression levels (HR=4.68 p=4e-05, log-rank test). (**G**) Survival analyses of KIAA0101 mRNA expression levels in HCC based on the Kaplan–Meier plotter database. Kaplan–Meier survival curves showed significant differences in OS between Caucasian HCC patients with high and low KIAA0101 mRNA expression levels (HR=1.74 p=0.018, log-rank test). Abbreviation: HCC: hepatocellular carcinoma. OS: overall survival.

To explore the prognostic significance of KIAA0101 in HCC cohorts, we performed prognostic analysis based on overall and different clinicopathological characteristics using the KM plotter [35]. Based on the results, increased mRNA expression of KIAA0101 was associated with shortened OS in all HCC patients (HR=2.09, p=4.1e-05) (Figure 4A).

When considering sex for subgroup analysis, HCC subjects with high KIAA0101 mRNA expression tended to have a shorter OS, regardless of whether they were male or female. However, the statistical effect of KIAA0101’s prognostic value for male HCC patients (HR=2.6, p=0.00021) (Figure 4B) appeared to be more powerful than that for female HCC patients (HR=1.87, p=0.03) (Figure 4C).

Furthermore, subgroup analysis of HCC patients was performed based on pathological staging. Patients with higher mRNA KIAA0101 expression had shorter OS than those with lower expression in both early (HR=2.34 p=0.00074) (Figure 4D) and advanced (HR=1.93, p=0.031) (Figure 4E) stages. Additionally, the prognostic effect of high mRNA expression of KIAA0101 tended to be stronger for early-stage HCC patients.

Finally, our analysis showed shorter OS for both Asian (HR=4.68 p=0.00004) (Figure 4F) and Caucasian (HR=1.74, p=0.018) patients (Figure 4G) with higher mRNA expression of KIAA0101 compared to those with lower expression, with the prognostic strength of KIAA0101 expression being greater in Asian patients.

### mRNA expression of KIAA0101 in HCC tumor and adjacent tissues

**Figure 5:**
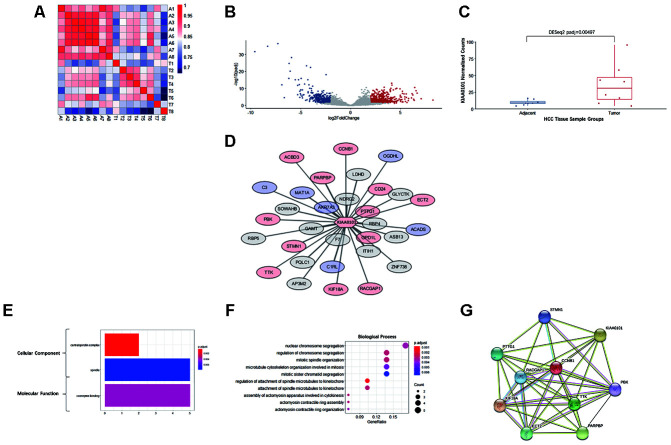
**RNA sequencing, differential analysis, transcriptomic coexpression, and functional analysis of our HCC specimens.** (**A**) Heatmap demonstrating the correlation between the 8 pairs of adjacent and tumor samples. A1-A8 are adjacent tissues; T1-T8 are tumor tissues. The more intense red indicates a higher correlation, and the more intense blue indicates the opposite. (**B**) The volcano plot showing the distribution of differentially expressed genes in KIAA0101. The log2FoldChange and adjusted p-value (padj) were calculated by R package DESeq2. Red dots indicate significantly upregulated genes (padj<0.05, long2FoldChange>2), and blue dots indicate significantly downregulated genes (padj<0.05, long2FoldChange< -2). (**C**) KIAA0101 mRNA expression levels in 8 pairs of HCC tumor and adjacent tissue samples. The normalized counts and adjusted p-value (padj) = 0.00497 were calculated by R package DESeq2. The boxplot demonstrates the normalized counts in individual samples (scattered dots), average normalized counts (midline of the box), upper quartile (upper edge of the box), and lower quartile (lower edge of the box). (**D**) The WGCNA correlation network including KIAA0101 and the top 31 coexpressing genes (weight>0.01). The red nodes represent 13 genes that are significantly upregulated (padj<0.05), and the blue nodes represent 6 significantly downregulated genes (padj<0.05). The length of edges connecting the nodes is in reverse proportion to the weighted correlation. (**E**) The results of GO analysis (cellular component and molecular function) of the top 31 coexpressing genes are shown in Figure 5F. The more intense red indicates higher significance, and the more intense blue indicates the opposite. The horizontal axis indicates the count of a specific term in the result of GO analysis. (**F**) GO analysis (biological process) of the top 31 coexpressing genes. The more intense red indicates a lower adjusted p-value and higher significance, and the more intense blue indicates the opposite. The horizontal axis indicates the ratio of a specific term count to the total count of terms. The size of the dot indicates the count of a specific term. (**G**) The STRING protein-protein interaction network involving 10 genes from the KIAA0101 coexpression network. The nodes represent the proteins encoded by the specific genes. Sky-blue edges: known interactions indexed by curated databases. Pink edges: experimentally determined known interactions. Green edges: predicted interactions based on gene neighborhoods. Black edges: coexpression relationships.

To further explore the mechanism of the prognostic value of KIAA0101, coexpression analysis was performed for eight pairs of our own HCC specimens and adjacent tissues. All 8 patients were HBV-positive Chinese males. As expected, the adjacent tissues, i.e., liver tissues, exhibited very strong correlations, whereas the tumor tissues displayed relatively weak correlations because of the tumor heterogeneity of HCC. Nonetheless, tumor and adjacent tissues from the same patient showed moderate correlations (Figure 5A). In addition, a subgroup of protein-coding genes was found to be differentially expressed in HCC, with an expected volcano-like distribution (Figure 5B), and KIAA0101 was among the significantly upregulated genes in HCC (DESeq2 adjusted p=0.00497) (Figure 5C).

### Coexpression and functional analysis of KIAA0101

Based on WGCNA analysis, the following 31 genes (correlation weight>0.01) are the tops genes coexpressed with KIAA0101: F7, AKR7A3, GAMT, GPD1L, NDRG2, ITIH1, RBP4, PARPBP, C1RL, PTTG1, MAT1A, PQLC1, STMN1, LDHD, CD24, KIF18A, SOWAHB, ASB13, AP3M2, RACGAP1, ZNF738, GLYCTK, PBK, CCNB1, ACADS, C3, TTK, ECT2, RBP5, ACBD3, and OGDHL (in descending order of weight). Among these KIAA0101-coexpressed genes, 12 were significantly upregulated with KIAA0101 and 9 significantly downregulated (Figure 5D).

GO analysis annotated the functions of the genes in the KIAA0101 coexpression module. The cellular component terms were enriched in the centralspindlin complex and spindle, which are structures associated with mitosis, and the centralspindlin complex was more significantly enriched with fewer gene counts (Figure 5E). The term coenzyme binding was enriched in the molecular function category (Figure 5E). For biological processes, the terms nuclear chromosome segregation (1 term of 5 counts), regulation of chromosome segregation, mitotic spindle organization, microtubule cytoskeleton organization involved in mitosis, mitotic sister chromatid segregation (4 terms of 4 counts), regulation of attachment of spindle microtubules to the kinetochore, attachment of spindle microtubules to the kinetochore (2 terms of 3 counts), assembly of actomyosin apparatus involved in cytokinesis, actomyosin contractile ring assembly and actomyosin contractile ring organization (3 terms of 2 counts) were enriched. Among these biological process terms, regulation of attachment of spindle microtubules to kinetochores was the most significantly enriched, and mitotic sister chromatid segregation was the least significantly enriched; enrichment of the other terms was of intermediate significance (Figure 5F).

Furthermore, 9 genes among the 31 KIAA0101-coexpressed genes were present in the protein-protein interaction network of KIAA0101, as determined by the STRING database, as follows: STMN1, PTTG1, CCNB1, RACGAP1, KIF18A, TTK, ECT2, PARPBP, and PBK (Figure 5G). Notably, KIAA0101 and these 9 coexpressed genes were observed to be upregulated in HCC. These interactions include experimentally determined interactions, interactions indexed by curated databases, gene neighborhoods, and coexpression relationships (Figure 5G).

### Expression of two KIAA0101 transcript variants in HCC

Previous studies have validated two major variants of KIAA0101 transcripts. Variant 1 (PCLAF-201, ENST00000300035.9) encodes a longer protein; variant 2 (PCLAF-202, ENST00000380258.6) encodes a shorter isoform because this transcript lacks exon 3 of variant 1 (ENSE00003784561), with a frameshift. Because these two protein isoforms exhibit a high affinity for different antibodies, it is important to evaluate their expression in HCC.

**Figure 6:**
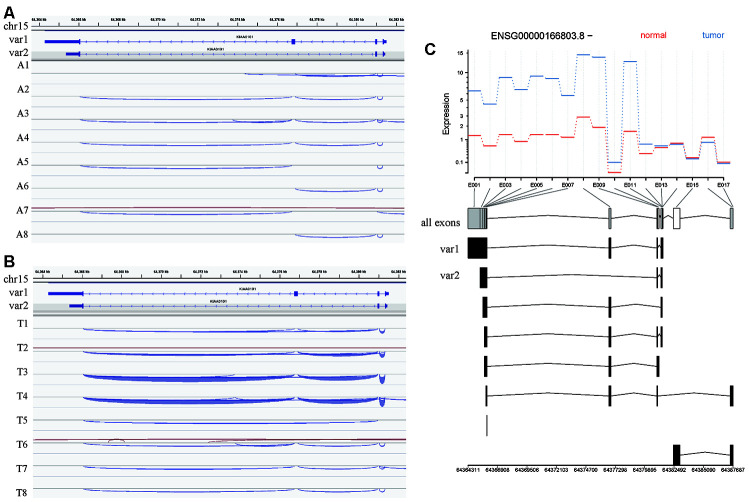
**Analysis of KIAA0101 transcripts variant 1 and variant 2 based on RNA sequencing of our HCC specimens.** (**A**) IGV visualization of splicing events in the genomic region covering the KIAA0101 transcript variants 1 and 2 in adjacent tissues. The top coordinate presents the location in the reference genome (GRch38), which ranges from 64,364 kb to 64,382 kb on chromosome 15. The exon usage of KIAA0101 transcripts variant1 and variant 2 is demonstrated below this coordinate. The splicing events in adjacent tissues A1-A8 are plotted and represented by curves that link the exons. The blue curve indicates splicing events on the minus strand, and the red curve indicates splicing events on the plus strand. (**B**) IGV visualization of splicing events in the genomic region covering the KIAA0101 transcript variants 1 and 2 in HCC tissues. The splicing events in HCC tissues T1-T8 are plotted. (**C**) Differential analysis of KIAA0101 (ENSG00000166803.8, - strand) exon usage in HCC and adjacent tissues. The red line indicates the average expression of different exons in the 8 normal tissue samples, and the blue line indicates those in the 8 tumor tissue samples. The horizontal axis demonstrates the position of all the exons from E001 to E017. The usage of these exons by different KIAA0101 transcripts is plotted below the horizontal axis, among which are variant 1 and variant 2. The bottom coordinate presents the corresponding reference genome coordinate.

Given that the main distinction between these two variants is exon 3 of variant 1, which is not present in variant 2, we speculated that the splicing pattern lacking this exon generates variant 2. One among 8 normal samples (Figure 6A) and 5 among 8 HCC samples (Figure 6B) harbored a splicing event that skips this exon.

Moreover, our alternative splicing analysis revealed no significant difference in exon usage for KIAA0101 between normal and HCC samples, including exon 3 of variant 1 (E008 among all exons). The exons included in variant 1 (E001-E014) were overall more highly expressed in HCC samples. In addition, the exons retained by processed noncoding transcripts (E016) had relatively higher expression in normal samples (Figure 6C).

## DISCUSSION

HCC ranks fifth among common solid tumors globally and second for cancer-associated mortality [36]. Novel and reliable biomarkers are needed to improve the clinical outcome of HCC patients. Large-scale genome analyses have indicated molecular features related to the OS of HCC [37–39] and novel biomarkers for early diagnosis, therapeutic monitoring and prognostic assessment. KIAA0101, a 15-kDa protein, was initially discovered by yeast two-hybrid screening in 2001 [10]. KIAA0101 functions by binding to PCNA [10], an essential scaffold molecule for DNA repair, replication, cell proliferation and tumor invasion [40]. In recent years, several studies [29, 30] have indicated that overexpression of KIAA0101 in peripheral blood serum or peripheral blood mononuclear cells is related to decreased OS in HCC and that KIAA0101 might function as a predictive biomarker for HCC with good sensitivity and specificity. There are also studies [31–33] demonstrating that KIAA0101 expression is significantly increased in HCC tissues compared with normal liver tissues and that patients with high expression usually have a poor prognosis. Additionally, there have been efforts to elucidate the reason for the correlation between KIAA0101 overexpression and poor prognosis. In vitro experiments have validated that KIAA0101 is targeted by Forkhead Box M1 (FoxM1) in HCC; this axis promotes the epithelial-mesenchymal transition (EMT) and thus metastasis in HCC [41]. These studies are the basis for the hypothesis that KIAA0101 is a potential prognostic biomarker for HCC. Nevertheless, these previous independent studies and the individual datasets in databases inevitably have sampling bias due to their small sample sizes and different patient populations. Moreover, the research methods and specimens used in different studies often vary. Therefore, the universality and clinical applicability of the conclusions of individual studies are not very strong. Regardless, when taken together, the diversity of research methods proves that the prognostic value of KIAA0101 in HCC is robust and reliable.

In this study, we focused on the factors that influence the prognostic strength of KIAA0101 and the underlying mechanisms. Firstly, we globally viewed KIAA0101 expression in cancer cell lines by using the CCLE database and found that levels were similar in liver cancers and in cancers in which KIAA0101 overexpression has been validated. We observed that the copy number and gene expression of KIAA0101 approximately correlated positively when liver cancer, glioma, lung non-small-cell cancer, pancreatic cancer, breast cancer, esophagus cancer, colorectal cancer, and kidney cancer datasets were combined. However, this correlation in the liver cancer dataset was not strong, as the samples with a low copy number of KIAA0101 showed equally high expression. Therefore, KIAA0101 is not likely to be a highly CNV-driven upregulated gene in HCC [42], and other driving forces are expected to play an important role in its overexpression. To study this correlation in detail, whole-exome sequencing (WES) or single-nucleotide polymorphism (SNP) array analysis combined with RNA-seq should be performed on HCC specimens.

Second, we investigated ONCOMINE, which involves large datasets of HCC transcriptome profiling generated by various high-throughput methods. Surprisingly, we found that the KIAA0101 level was associated with virus infection status and tumor grade. The Chen liver dataset showed higher KIAA0101 expression in HCV-positive HCC than in HBV-positive HCC, with the lowest level in no-value samples. It should be noted that the HCV-positive HCC dataset (4 samples) was smaller than the HBV-positive dataset (90 samples); the small size of the HCV-positive group makes it less representative. Furthermore, based on the Wurmbach liver dataset [31], KIAA0101 is expressed at a higher level in high-grade HCC samples, indicating that KIAA0101 is also a potential biomarker for HCC grading. Further studies should investigate the KIAA0101 expression level in HCC of different stages in a larger cohort.

Third, by using KM plotter, we identified that sex, tumor stage, and ethnicity influence the prognostic significance of KIAA0101 in HCC. For example, the OS of patients harboring high KIAA0101 mRNA expression was significantly reduced, which was consistent with the conclusion of previous studies [29, 30]. The clinical prognostic value of KIAA0101 in HCC patients was not impaired by sex, clinicopathological stage, or ethnicity; instead, its prognostic strength appeared to be more potent in male patients than in female patients, in early-stage patients than in advanced-stage patients, and in Asian patients than in Caucasian patients. There is no prior evidence for this sexual difference, and further research is needed. Regarding tumor stage, a study conducted by Yuan et al [43] reported that HCC patients with KIAA0101 overexpression were two times more likely to have advanced-stage disease than those with normal expression, as confirmed in Abdelgawad’s study [29]. Because sex, tumor stage and ethnicity influence the prognostic significance of KIAA0101 in HCC patients, the potential predictive model for HCC patient prognosis based on KIAA0101 should consider these three factors. To validate such a predictive model, a multicenter clinical study of the correlation between KIAA0101 expression level and HCC patient prognosis should involve careful subgrouping according to these three factors.

In particular, ethnic differences are of great interest. Indeed, it was found for the first time that the prognostic strength of KIAA0101 for HCC is significantly higher in the Asian population than in the Caucasian population, indicating a good clinical application potential of KIAA0101 as a prognostic biomarker in the former, which bears a high incidence of HCC. Regarding another HCC high-risk population, Black individuals, we were unable to assess the prognostic value of KIAA0101 because there were only 17 patients in the database. Therefore, in the future, more multicenter, large-scale, and prospective clinical cohort studies are needed to explore the universality of KIAA0101 as a prognostic biomarker in HCC patients. Moreover, it is widely acknowledged that the prevalence of HBV-induced HCC is significantly higher in Asian developing countries such as China but that HCV-induced HCC is more prevalent in the Caucasian population [31]. Thus, we hypothesized that HBV and HCV infections are associated with the mechanism underlying the process by which KIAA0101 contributes to the shorter OS of patients.

Accordingly, we performed coexpression analysis of KIAA0101 using our own specimens to explore the underlying mechanisms. To control diversity among the samples, we recruited 8 HBV-positive Chinese male patients. The protein encoded by KIAA0101 is a chromatin- and protein-binding molecule that functions in DNA replication, the centrosome cycle and regulation of the cell cycle and is involved in the structure of the centrosome and nucleus. Hence, it is reasonable that KIAA0101-coexpressed genes are mainly associated with mitosis, cytoskeleton construction, and chromosome segregation. The data indicate that KIAA0101 and its coexpressed genes play an important role in cell cycle and tumor cell replication. Among the 31 coexpressed genes, specifically 12 significantly regulated genes, 9 were verified by the STRING database to be involved in protein-protein interactions. Among these 9 genes, PTTG1 (PTTG1 Regulator of Sister Chromatid Separation, Securin) participates in the virus infection pathway, and STMN1 (Stathmin 1) is related to the response to the virus. A shortcoming of our results is that no HCV-positive or hepatitis virus-negative HCC specimens were involved in the coexpression analysis because the majority of hepatitis virus-infected HCC patients in our medical center are HBV positive, with few being HCV positive. Comparative studies of KIAA0101 and coexpressed gene levels in HBV-infected or HCV-infected HCC patients of the same ethnicity and sex using the identical RNA-seq platform and analysis pipeline will help to elucidate the mechanism that links hepatitis virus infection status, KIAA0101 expression level, and HCC oncogenesis.

Through a review of the related literature, we identified a complex regulatory network between KIAA0101 (also known as NS5ATP9) and HCV-related HCC. With KIAA0101 as the center, this regulatory network involves upstream molecules NF-kB and NS5A, downstream molecules Beclin 1, and the downstream p53-p21, MEK/ERK, PIK3C3/VPS34 pathways and is closely related to autophagy.

NS5A encodes a 477-aa phosphoprotein harboring differently phosphorylated forms of 56 and 58 kDa in size with modified serine residues, which is likely to have interaction with various cellular proteins and involved in modulation of cellular signaling pathways, cell growth and pathogenesis of HCV [44–46]. HCV NS5A has been previously revealed to upregulate NS5ATP9 via binding to the promoter, a minimal promoter region within 211 bp (nucleotides 161 to +50 bp) immediately upstream of the transcriptional initiation site [47]. Thus, NS5ATP9 can be up-regulated by NS5A-upregulated to be involved in the pathogenesis of HCV-related HCC. The interaction between NS5ATP9 and PCNA is likely to be competitively suppressed by p21 [10], and the p53–p21 pathway (critically regulating DNA replication) might rigorously modulate the expression and function of NS5ATP9 [25]. Exogenous overexpression of NS5ATP9 is capable of promoting tumor cell proliferation and transforming NIH3T3 cells in vivo. Additionally, NS5ATP9 silencing could significantly inhibit cell growth [11, 25]. Nevertheless, opposite findings were reported by Wang Q et al [48], who revealed that overexpression of NS5ATP9 suppressed Bel7402 cell proliferation, while RNAi-mediated knockdown of NS5ATP9 enhanced HepG2 cell growth. In cases of HCV NS5A expression, suppressed proliferation of HepG2 cells may be reversed by RNAi-mediated targeting of NS5ATP9, indicating that NS5ATP9 might function as an anti-proliferative gene to be involved in inhibiting HCV NS5A-mediated cell growth through the MEK/ERK signaling pathway.

NF-kB, also known as Rel/NF-kB, consists of a group of transcription factors with evolutionary conservation, which participate in responses to environmental stimuli [49]. NF-kB is highly structurally conserved, from sea anemones to humans. NF-kB has been widely revealed to be critically involved in apoptosis, cell cycle regulation, and oncogenesis [50, 51], meanwhile, the increased NF-kB activity cancer cells has been considered to play a vital role in maintaining their survival [52]. The increased expression of this factor has been reported in several types of cancer, including colon, thyroid, breast and bladder. In addition, several chromosomal changes of NF-kB family (c-Rel, p52, and p65) are located within breakpoint regions related to the pathogenesis of certain diseases, such as leukemia as well as non-Hodgkin’s lymphoma [52, 53]. Li K et al [54] revealed that NF-kB (p50) could bind with the NS5ATP9 promoter as a new interaction molecular partner of NS5ATP9; notably, the binding region was contained within 156 bp (nucleotides 5 to 161) immediately upstream of the transcription initiation site. Collectively, the above outcomes implicate the involvement of NF-kB in regulating NS5ATP9 gene expression during tumorigenesis. Thus, NS5ATP9 might be rigorously controlled by a complicated network of interactors, including p53, NF-kB, NS5A, etc.

Increasing evidence has potently indicated that autophagy can aggravate carcinogenesis and protect tumor cells from death [55]. Beclin 1, a molecule acting on upstream of autophagosome formation, determines the autophagy process via the regulation of PIK3C3/VPS34 activity [56] as well as by subsequently recruiting additional ATG proteins for initiating autophagosome formation [57]. The expression of NS5ATP9 is associated with several types of malignancies, which also participates in regulating autophagy evoked by HCV NS5A protein. By using luciferase-reporter assay, Quan M [58] demonstrated that both NS5ATP9 and NS5A can transactivate the promoter activity of Beclin 1, while NS5A failed to transactivate the Beclin 1 promoter after NS5ATP9 silencing in HepG2 and L02 cells. Similarly, NS5ATP9 knockdown could attenuated the up-regulated mRNA and protein expression of Beclin 1 by NS5A. Moreover, elevated accumulation of vacuoles carrying the autophagy marker LC3 were detected in HepG2 and L02 cells with transient overexpression of NS5ATP9, which was consistent to the conversion of endogenous LC3-I to LC3-II. On the contrary, the conversion of endogenous LC3-I to LC3-II was not increased by NS5A in HepG2 cells after NS5ATP9 silencing. Taken together, these outcomes highlight a vital potential role of NS5ATP9 in hepatocyte autophagy induced by HCV NS5A. In another research to explore the involvement of NS5ATP9-mediated autophagy in cancer cell growth, Quan M et al [59] demonstrated the necessity of NS5ATP9-mediated autophagy in promoting cancer cell proliferation. NS5ATP9-triggered autophagy relied on the upregulation of Beclin 1 under starvation situation, and the promoter activity of Beclin 1 was not increased in starved NS5ATP9-silenced cells. Therefore, autophagy suppression might be effective to inhibit the proliferation of tumors with NS5ATP9 overexpression.

Although increased expression of KIAA0101 mRNA has been validated in HCC, the amount of protein encoded by KIAA0101 is not necessarily upregulated. As a potential biomarker, if the KIAA0101 protein is overexpressed in HCC tissue and consistent with mRNA expression, protein-level methods such as immunohistochemistry (IHC) can be used to quantify KIAA0101 in HCC tissues.

It has been reported that variant 1 of KIAA0101 is overexpressed in HCC tissue [60] but that variant 2-encoded proteins are upregulated in adjacent tissues [61]. According to the RNA-seq results for our specimens, both variant 1 and variant 2 were elevated in HCC tissues compared with adjacent tissues. Moreover, the results of alternative splicing analysis indicated no significant difference in KIAA0101 exon usage between tumor and normal tissues. Indeed, the alternative splicing analysis based on next-generation RNA-seq is affected by fragmented transcripts due to the limitations of read lengths. Although the frequency of capturing a specific splicing event tends to reveal the frequency of its occurrence, less frequent splicing events are likely to not be captured if expression of the parental gene is low. In general, the alternative splicing analysis based on third-generation sequencing might be helpful for more precisely unravelling the expression and prognostic value of each variant.

## CONCLUSION

We combined large datasets in the CCLE, ONCOMINE, and KM plotter databases and performed coexpression and alternative splicing analyses using our specimens. The large datasets of transcriptome profiling in CCLE and ONCOMINE demonstrate KIAA0101 overexpression in HCC. KIAA0101 has the potential to serve as a novel prognostic biomarker for HCC, and its prognostic value differed regarding sex, tumor stage, and ethnicity. KIAA0101 is unlikely to be a highly CNV-driven upregulated gene in HCC, and other driving forces, such as HBV and HCV infections, are likely to be associated with the mechanism underlying the process by which KIAA0101 contributes to shorter OS in HCC patients.

## MATERIALS AND METHODS

### CCLE database analysis

The online database Cancer Cell Line Encyclopedia (CCLE: https://portals.broadinstitute.org/ccle/), a compilation of enormously parallel sequencing, copy number and gene expression data for 947 human cancer cell lines, was used to determine the gene expression and copy number of KIAA0101 in liver cancer, glioma, lung nonsmall-cell cancer, pancreatic cancer, breast cancer, esophageal cancer, colorectal cancer, and kidney cancer.

### ONCOMINE database analysis

ONCOMINE (http://www.oncomine.org), a publicly accessible online cancer microarray database, was employed to detect KIAA0101 mRNA expression differences between cancer tissues and normal tissues in different types of malignancies, including HCC. Student’s t-test was applied to compare datasets for tumor and normal control samples. The thresholds were set as follows: p-value, 0.01; fold change, 2; gene rank, 10%; analysis type, cancer vs. normal analysis; gene, KIAA0101; data type, mRNA; sample type, clinical specimen cancers. Genes, datasets, sample sizes, fold change, t-test, and p-value were acquired from analyses with statistical significance. Four datasets involving HCC were included in the meta-analysis: the Wurmbach liver [31], Chen liver [32], Roessler liver [33], and Roessler liver 2 [33] datasets. Furthermore, expression of KIAA0101 in HCC samples with different virus infection statuses was assessed by analyzing the Chen liver dataset [32]; that in HCC samples of different tumor grades was investigated using the Wurmbach liver dataset.

### KM plotter survival analysis

We applied the KM plotter (http://kmplot.com/analysis/) to perform survival analysis to evaluate the prognostic values of KIAA0101 in HCC patients. HCC patients were categorized into high and low expression groups according to the median value of KIAA0101 mRNA expression. KM survival curves, log-rank p-values, and HRs with 95% CIs were calculated and plotted on the KM plotter webpage. A p-value < 0.01 indicated statistical significance. We also subgrouped HCC patients according to sex, ethnicity, and tumor staging.

### RNA sequencing and differential analysis of HCC specimens

This study was approved by the Ethics Committee of Peking Union Medical Hospital. Surgically removed HCC specimens were collected from 8 patients who provided informed consent. The detailed baseline data for these 8 patients are shown in Table 1. The specimens were validated as HCC by pathological reports. cDNA library preparation and next-generation RNA sequencing (Illumina) were performed for the 8 pairs of tumor and adjacent tissue samples according to a standard protocol. The software fastp was applied for quality control of these RNA-seq data and the removal of low-quality reads, adapter sequences, and the tail and head of raw reads. STAR software was then used to map the clean reads to the human genome (GRCh38 reference genome and gencode v21 annotation), followed by counting the reads by using featureCounts software. These raw counts were processed by the R package DESeq2 to determine the fold change (log2FoldChange) and significance (adjusted p-value, padj) of the genes differentially expressed between the tumor and adjacent tissue samples. Normalized counts were used to calculate the correlation between different samples, and the correlations are represented as a heatmap. Normalized counts were also used to draw a boxplot of KIAA0101 in HCC and normal samples. A volcano plot presents the log2FoldChange and padj values of differentially expressed genes between the HCC and normal samples.

**Table 1:** The baseline data for 8 HCC patients recruited for RNA sequencing.

**Baseline characteristics**	**n(%)**
Gender	
Male	8 (100.0)
Female	0 (0.0)
Age	
≤50	6 (75.0)
>50	2 (25.0)
BMI	
≤18.5	0 (0.0)
18.5-24.9	6 (75.0)
>24.9	2 (25.0)
HBV	
Positive	8 (100.0)
Negative	0 (0.0)
HCV	
Positive	0 (0.0)
Negative	8 (100.0)
CA199	
≤10	3 (37.5)
>10	5 (62.5)
Preoperative GGT (μ/L)	
≤40	1 (12.5)
40-200	5 (62.5)
>200	2 (25.0)
Bilirubin (umol/L)	
≤20	6 (75.0)
>20	2 (25.0)
Albumin (g/L)	
≤40	2 (25.0)
>40	6 (75.0)
Differentiation of HCC	
Low	1 (12.5)
Low-Intermediate	4 (50.0)
Intermediate	2 (25.0)
High	1 (12.5)
HCC-related syndromes	
Cirrhosis	8 (100.0)
Jaundice	2 (25.0)

### Transcriptomic coexpression and functional analysis

The R package WGCNA (Weighted Correlation Network analysis) was used to identify the coexpression gene module involving KIAA0101, with correlations weighted over 0.01 plotted using Cytoscape software. Gene Ontology analysis was performed to examine the annotation and enrichment of the coexpressing genes in the categories biological process, molecular function, and cellular component. The online software STRING (http://string-db.org/cgi/input.pl) was utilized to validate protein-protein interactions between KIAA0101-coexpressing genes.

### Alternative splicing analysis

Integrative Genomics Viewer (IGV) software was used to visualize splicing events in the genomic region covering the KIAA0101 transcript variants 1 and 2. The GRCh38 reference genome, Gencode v21 annotation, and bam file generated by STAR software were inputted into IGV, which processed and demonstrated the splicing events automatically.

The software featureCounts was applied to prepare annotations for exons by processing gencode v21 annotation and to count the exon usage of the reads generated by STAR. The R package DEXSeq was then used to visualize the differential exon usage of KIAA0101 in tumor tissues and normal tissues.

### Ethical statement

This study was approved by the Ethics Committee of Peking Union Medical Hospital. Surgically removed HCC specimens were collected from 8 patients who provided informed consent. The authors are accountable for all aspects of the work in ensuring that questions related to the accuracy or integrity of any part of the work are appropriately investigated and resolved.
